# Comparison of Early Complication Rates After High Tibial Osteotomy Versus Unicompartmental Knee Arthroplasty for Knee Osteoarthritis

**DOI:** 10.1177/23259671231219975

**Published:** 2024-01-04

**Authors:** Theofilos Karasavvidis, Nathan P. Fackler, Kylie T. Callan, Brandon E. Lung, Dean Wang

**Affiliations:** † Department of Orthopaedic Surgery, University of California, Irvine, Orange, California, USA; ‡ Department of Orthopaedic Surgery, Hospital for Special Surgery, New York, New York, USA; § Department of Orthopaedic Surgery, University of California, San Diego, San Diego, California, USA; ‖ Department of Biomedical Engineering, University of California, Irvine, Irvine, California, USA; Investigation performed at University of California, Irvine, Irvine, California, USA

**Keywords:** osteoarthritis, high tibial osteotomy, unicompartmental knee arthroplasty, complications

## Abstract

**Background:**

Although both high tibial osteotomy (HTO) and unicompartmental knee arthroplasty (UKA) can be utilized to treat unicompartmental osteoarthritis (OA) in select patients, the early complication rates between the 2 procedures are not well understood. Understanding of the complication profiles for both procedures would help clinicians counsel patients with unicompartmental knee OA who may be eligible for either treatment option.

**Purpose:**

To compare the 30-day complication rates after HTO versus UKA for the treatment of knee OA using the American College of Surgeons National Surgical Quality Improvement Program (NSQIP) database.

**Study Design:**

Cohort study; Level of evidence, 3.

**Methods:**

NSQIP registries between 2006 and 2019 were queried using Current Procedural Terminology codes to identify patients undergoing HTO and UKA for knee OA. Patients >60 years of age were excluded. Patient demographics, preoperative comorbidities, and intraoperative data were collected. Postoperative 30-day complications, including venous thromboembolism (VTE), urinary tract infection (UTI), transfusion, surgical-site infection (SSI), and reoperations were recorded. Complication rates between treatment groups were compared using a multivariate logistic regression model adjusted for sex, age, body mass index, steroid use, respiratory status (smoking/dyspnea/chronic obstructive pulmonary disease), diabetes, and hypertension.

**Results:**

A total of 156 patients treated with HTO and 4755 patients treated with UKA for knee OA were identified. Mean patient ages were 46 years for the HTO group and 53.4 years for the UKA group. Operative time was significantly longer in the HTO group versus the UKA group (112 minutes vs 90 minutes; *P* < .001). Multivariate analyses found no significant differences in VTE (1.3% vs 0.6%), UTI (0.6% vs 0.3%), transfusion (0.6% vs 0.2%), deep SSI (0.6% vs 0.1%), and reoperation (1.3% vs 1%) rates between HTO and UKA groups. The HTO group had a higher rate of superficial SSI compared with the UKA group (2.6% vs 0.6%; *P* = .006) (adjusted odds ratio, 4.2; 95% CI, 1.4-12.5; *P* = .01).

**Conclusion:**

There were no differences in 30-day VTE, UTI, transfusion, deep SSI, and reoperation rates for HTO versus UKA in the treatment of knee OA. HTO was associated with a higher rate of superficial SSI compared with UKA. These findings serve to guide clinicians in counseling patients regarding the early risks after HTO and UKA.

Knee osteoarthritis (OA) is a leading cause of debilitating pain and is characterized by cartilage damage, inflammation, and progressive loss of knee function.^1^ Even though OA may affect any 1 or all 3 compartments of the knee, unicompartmental OA occurs in up to one-third of knee OA patients.^3,18,41^ The medial tibiofemoral compartment of the joint is more commonly affected compared with the lateral or patellofemoral compartments.^18,40^ Both high tibial osteotomy (HTO) and unicompartmental knee arthroplasty (UKA) are established treatment methods for medial unicompartmental knee OA, despite having different philosophies.^9^

Unicompartmental OA is often caused by limb malalignment, which may occur due to repetitive asymmetric loading across the physis, as is commonly observed in athletes.^5,26,32^ HTO was introduced to correct limb malalignment, unload the affected compartment, preserve the native articular cartilage, and potentially delay the need for arthroplasty.^33,34^ Depending on the location and extent of the deformity, HTO can redistribute the mechanical loads to the nonaffected compartment via multiple techniques, including opening-wedge HTO, closing-wedge HTO, and dome osteotomies.^14,44^ However, early complications associated with HTO can include neurovascular injury, fracture, nonunion, and venous thromboembolism (VTE).^14,24^

UKA, first introduced in the 1970s as an alternative to total knee arthroplasty or HTO, is a partial joint resurfacing procedure in which an implanted prosthesis is utilized to treat the affected compartment while the nonaffected compartment is preserved.^11,44^ UKA offers patients a less invasive surgery with preservation of ligaments and knee kinematics and potentially faster recovery time compared with HTO due to allowance of immediate full weightbearing.^11^ However, potential early shortcomings associated with UKA include periprosthetic infection, VTE, and inaccurate positioning of the implant component without correction of malalignment.^16^

Although both HTO and UKA can be utilized to treat unicompartmental OA in select patients, the early complication rates between the 2 procedures are still not well understood.^6,28,43^ Understanding of the complication profiles for both procedures would help clinicians counsel patients with unicompartmental knee arthritis who may be eligible for either treatment option. Therefore, the purpose of the current study was to compare the 30-day perioperative complication rates for HTO and UKA for the treatment of knee OA using a large national database (American College of Surgeons National Surgical Quality Improvement Program [NSQIP]). We hypothesized that there would be no differences in 30-day perioperative complication rates between patients who underwent HTO or UKA after controlling for patient demographics and comorbidities.

## Methods

### Data Source

The data for this study were retrospectively collected from NSQIP database. The NSQIP database is a prospectively collected, risk-adjusted, outcomes-based program with >500 participating institutions in the United States. The 2019 version of the database was used, which contains >300 variables including preoperative risk factors, intraoperative variables, and 30-day postoperative complications for patients undergoing major surgical procedures. The database is maintained and updated by trained clinical reviewers, who abstract patient information from patient interviews, medical records, and operative reports through the 30th postoperative day, regardless of discharge.^4^ This study was exempt from institutional review board approval, as the database was already de-identified of all patient health information. The study was conducted according to the Strengthening the Reporting of Observational Studies in Epidemiology guidelines.^38^

### Patient Population

In this retrospective comparative study, patients who underwent HTO or UKA for knee OA between January 1, 2006, and December 31, 2019, were identified using the Current Procedural Terminology (CPT) codes 27455 and 27457 for HTO and 27446 for UKA. Patients were excluded if they were >60 years of age or if HTO or UKA was performed for a diagnosis other than OA of the knee (International Classification of Diseases [ICD], Ninth Revision, codes 715.16, 715.36, 715.96; ICD, Tenth Revision, codes M17.0, M17.9, M17.11, M17.12).

Patient demographics, including sex, age, body mass index (BMI), and history of comorbidities, including diabetes, smoking, dyspnea, chronic obstructive pulmonary disease (COPD), functional status, congestive heart failure (CHF), hypertension (HTN), renal failure, dialysis, steroid use, and bleeding disorders, were extracted for analysis. Concomitant procedures, including cartilage restoration (CPT codes 29886, 29887, 27416, 27415, 27412), meniscal allograft transplantation (CPT code 29868), cruciate ligament repair/reconstruction (CPT codes 29888, 29889), arthroscopic chondroplasty (CPT code 29877), and arthroscopic meniscal treatment (CPT codes 29880, 29881, 29882, 29883) in the HTO group were identified. Operative time, postoperative length of hospital stay (LOS), and reoperation within 30 days of the index surgery were also collected. In the NSQIP database, operative time is defined as the total operation time in minutes, while LOS is defined as the LOS after operation to discharge. Thirty-day complications of interest included pulmonary embolism (PE), VTE (deep venous thrombosis [DVT] or PE), urinary tract infection (UTI), need for transfusion, superficial surgical-site infection (SSI), and deep SSI.

### Statistical Analysis

Continuous variables were described with mean ± SD, whereas categorical variables were reported with absolute and relative frequencies.^39^ The *t* test was conducted to compare continuous variables, while binary outcomes were compared using the chi-square or Fisher exact test as appropriate. Univariate and multivariate logistic regression models were developed to compare complication rates between HTO and UKA. The following variables were decided a priori to be included in the multivariate model: sex, age, steroid use, and respiratory status (smoking/dyspnea/COPD). After analyzing patient characteristics, additional variables were added in the multivariate model, when significant differences were identified between the groups. The threshold for statistical significance was *P* < .05. Stata 17 (StataCorp LLC) was used for all analyses.

## Results

### Patient Characteristics

**Figure 1.**
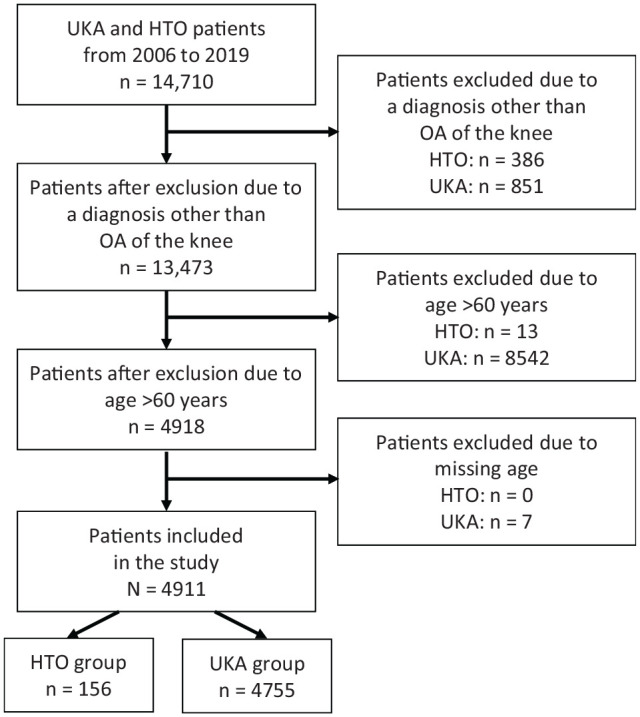
Flowchart of patient inclusion in the study. HTO, high tibial osteotomy; OA, osteoarthritis; UKA, unicompartmental knee arthroplasty.

A total of 156 patients treated with HTO and 4755 patients treated with UKA were identified (Figure 1). Table 1 shows the demographic characteristics, preexisting comorbidities, and LOS of all 4911 patients included in the final analysis. The 2 groups were statistically similar in terms of smoking, dyspnea, functional status, CHF, renal failure, dialysis, steroid use, bleeding disorders, and LOS. Compared with the HTO group, the UKA group had more female patients (53.3% vs 28.8%; *P* < .001), older patients (53.4 vs 46 years; *P* < .001), more patients with diabetes (11.2% vs 3.2%; *P* = .002), patients with higher BMI (33.2 vs 31.7; *P* = .003), and more patients with HTN (42.3% vs 22.4%; *P* < .001). There were no concomitant cruciate ligament reconstructions, meniscal allograft transplantations or meniscal repairs performed in the HTO group. Three patients (1.9%) in the HTO group underwent concomitant cartilage repair procedure (1 autologous chondrocyte implantation, 2 osteochondral allograft transplantation), 12 patients (7.7%) received concomitant chondroplasty, and 23 patients (14.7%) underwent concomitant meniscectomy.

**Table 1:** Baseline Demographic and Clinical Characteristics^*a*^

Variable	HTO (n = 156)	UKA (n = 4755)	*P*
Female sex	45 (28.8)	2535 (53.3)	<.001
Age, y	46 ± 8.4	53.4 ± 5.5	<.001
BMI	31.7 ± 5.9	33.2 ± 6.6	.003
Diabetes	5 (3.2)	531 (11.2)	.002
Smoking	20 (12.8)	780 (16.4)	.2
Dyspnea	2 (1.3)	128 (2.7)	.3
Functional health status prior surgery			.7
Independent	155 (99.4)	4668 (98.2)	
Dependent	1 (0.6)	87 (1.8)	
COPD	0	84 (1.8)	.09
CHF in 30 d before surgery	0	5 (0.1)	.7
HTN	35 (22.4)	2013 (42.3)	<.001
Renal failure	0	1 (0.02)	.8
Dialysis	0	2 (0.04)	.8
Steroid use	2 (1.3)	72 (1.5)	.8
Bleeding disorders	0	48 (1)	.2
Length of hospital stay, d	1.3 ± 1	1.4 ± 1.5	.4

a Data are reported as mean ± SD or n (%). BMI, body mass index; CHF, congestive heart failure; COPD, chronic obstructive pulmonary disease; HTN, hypertension; HTO, high tibial osteotomy; UKA, unicompartmental knee arthroplasty.

### Outcomes and Complications

Operative time was significantly longer in the HTO group versus the UKA group (mean time, 112 vs 90 minutes; *P* < .001). The absolute numbers and rates of all 30-day postoperative complications are shown in Table 2. The rates for all complications were <2.7%. No event of PE was observed in the HTO group, while a 0.2% PE rate was demonstrated in the UKA group. Univariate analysis found no significant differences between the HTO and UKA group in VTE (1.3% vs 0.6%), UTI (0.6% vs 0.3%), transfusion (0.6% vs 0.2%), deep SSI (0.6% vs 0.1%), and reoperation (1.3% vs 1%) rates. In the multivariate logistic regression analysis, which controlled for sex, age, BMI, steroid use, respiratory status (smoking/dyspnea/COPD), diabetes, and hypertension, the differences in complication rates remained insignificant. Univariate analysis revealed that patients undergoing HTO had a higher rate of superficial SSI compared with those undergoing UKA (2.6% vs 0.6%; *P* = .006). This difference was also demonstrated in the multivariate analysis (adjusted odds ratio, 4.2; 95% CI, 1.4-12.5; *P* = .01).

**Table 2:** 30-Day Postoperative Complications^*a*^

Outcome	HTO (n = 156)	UKA (n = 4755)	Univariate Analysis		Multivariate Analysis	
	n (%)	n (%)	OR (95% CI)	*P*	Adjusted OR (95% CI)	*P*
PE	0	9 (0.2)	1		1	
VTE	2 (1.3)	29 (0.6)	0.47 (0.1-2)	.3	0.44 (0.1-1.94)	.3
UTI	1 (0.6)	14 (0.3)	0.46 (0.6-3.5)	.4	0.28 (0.03-2.21)	.2
Transfusion	1 (0.6)	12 (0.2)	0.39 (0.5-3.03)	.4	0.27 (0.04-2.14)	.2
Reoperation	2 (1.3)	48 (1)	0.78 (0.19-3.26)	.7	0.59 (0.18-1.96)	.4
SSI						
Superficial	4 (2.6)	28 (0.6)	0.22 (0.08-0.65)	.006	0.24 (0.08-0.71)	.01
Deep	1 (0.6)	5 (0.1)	0.16 (0.02-1.4)	.1	0.14 (0.01-1.29)	.08

a HTO is the reference group for the univariate and multivariate analyses. HTO, high tibial osteotomy; OR, odds ratio; PE, pulmonary embolism; SSI, surgical-site infection; UKA, unicompartmental knee arthroplasty; UTI, urinary tract infection; VTE, venous thromboembolism.

## Discussion

Using a large surgical database, the 30-day complication profiles of HTO versus UKA in 4911 patients were calculated and compared. This study demonstrated no statistically significant differences in rates of VTE, UTI, transfusion, deep SSI, and reoperation between patients treated with HTO versus UKA for knee OA. HTO was associated with a higher rate of superficial SSI compared with UKA in both univariate and multivariate analyses. All complication rates were <2.7%.

Both HTO and UKA are well-established treatments for unicompartmental knee OA.^29^ Studies that have directly compared the 2 treatment modalities are scarce. Some groups have reported that UKA has fewer complications than HTO,^9,29^ while other researchers have demonstrated no significant differences in complication rates between the 2 procedures.^13,42^ In a study of 70,464 patients aged between 50 and 70 years from the Korean National Health Insurance claims database, HTO showed a higher risk of reoperation than UKA at 10-year follow-up, while postoperative DVT and SSI were significantly higher in UKA than in HTO.^19^ A retrospective comparative study including patients from the Military Health System also demonstrated that HTO was associated with a higher reoperation rate when compared with UKA.^27^ Stukenborg-Colsman et al^36^ conducted one of the few relevant randomized controlled trials that can be identified in the literature, comparing 32 patients treated with HTO and 28 patients treated with UKA, and concluded that HTO was associated with more intraoperative and postoperative complications. However, the small sample size limits the generalizability of this study. Additionally, the study was performed in 2001, and there have been improvements in the surgical techniques and implants for both procedures.^29^ The present study, which includes a total of 4911 patients between 2006 and 2019, demonstrated no significant differences between the groups regarding 30-day all-cause reoperation, VTE, UTI, transfusion, and deep SSI, while superficial SSI was higher in the HTO group. Even though no causal relationship was identified between a specific factor and the superficial SSI events, the longer operative time as a consequence of a concomitant surgical procedure might have increased the likelihood of SSI in this patient group.

Some studies have examined the incidence of PE following UKA. Ogonda et al^22^ analyzed data from the National Joint Registry for England and Wales and found a 1.2% PE rate after UKA, while the Humana database has demonstrated a PE rate of 0.8% after UKA.^2,22^ Schneider et al^30^ assessed that same database as the present study, without applying an age limit in the UKA group, and found a lower PE rate (0.2%) after UKA. The results of the current study align with the findings by Schneider et al and suggest that even though PE is an infrequent complication, prophylaxis should be considered to avoid devastating consequences. The rates of PE and VTE after HTO are not well-documented in the literature, with VTE reports ranging from 0.4% to 2% and DVT up to 16%.^7,23,25^ The potential reason for this substantial variation of reported results between studies is the difference in the methods and study designs.^20,37^ No events of PE were observed in the present study, while 1.3% of the HTO group presented with VTE within 30 days.

To clarify current ambiguities that arise from smaller cohort studies, several groups have attempted to pool data and conduct a systematic review and meta-analysis comparing HTO and UKA. Han et al^9^ performed a meta-analysis, including 16 studies, and demonstrated no significant difference between HTO and UKA in terms of reoperation. In this study, HTO had a higher complication rate than did UKA. Another meta-analysis analyzing data from 6193 patients demonstrated satisfactory knee function results for both surgical techniques and fewer primary complications in the UKA group. Primary complications included infection, DVT, peroneal nerve palsy, and pain, among others.^3^ Fu and colleagues^8^ pooled data from 11 comparative studies and found no significant differences in rates of complication. However, the authors highlighted the limitation of available randomized trials and the variation in the techniques used for both procedures.

Surgeons have traditionally favored HTO when dealing with young physically active patients and UKA in cases of older individuals with less physical activity requirements.^9,10,29,35^ HTO has been associated with quicker return to sports activities and superior range of motion compared with UKA both in primary comparative studies and in meta-analyses.^3,12,29^ Even after excluding patients >60 years of age, mean patient ages in the current study were significantly different between the 2 groups (46 years for the HTO group and 53.4 years for the UKA group), confirming that surgeons in the United States tend to perform HTO in younger patient populations. However, surgeons in some countries do not have access to UKA treatments and can only perform HTO in the early knee OA population. Based on the current literature, it is unclear whether there should be a strict age cutoff for HTO. Although older age has been associated with worse survivorship after HTO in some studies, HTO may still be a valid option for older active patients with unicompartmental knee OA.^15^ When making the decision to perform surgery on older patients who might be suitable for HTO, it is important to take into account additional risk factors. Instead of focusing solely on a person’s biological age, it may be more effective to consider their physiological age and the severity of their disease when predicting the likelihood of a revision.^12,31^ The results of this current study can aid physicians in counseling patients on the early perioperative risks of HTO, which should also go along with counseling them on the risk of conversion to arthroplasty.

### Limitations

NSQIP has uniform standards for the reporting of adverse events and undergoes interreliability audits that make it a highly reliable resource.^4^ Nevertheless, the data can be subject to errors in coding or underreporting. Second, there were only 156 HTO patients compared with 4755 UKA patients, with many HTO patients excluded due to a nonknee OA diagnosis. This discrepancy in the number of patients per group may have led to type II errors. Type II errors occur when the study fails to detect a significant difference or effect that actually exists in the population. In this case, the small sample size of the HTO group and the exclusion of certain patients may have limited the study’s ability to identify true differences between HTO and UKA complications. Consequently, it is possible that the study overlooked significant associations or effects related to HTO due to insufficient statistical power, leading to a type II error. Besides, this discrepancy led to the final sample size’s having <0.8 power to detect a significant difference in terms of the complications assessed. Thus, the head-to-head comparative results should be interpreted with caution. Third, patients are only tracked during the first 30 days after surgery, and the database does not report orthopaedic-specific complications, such as fracture and neurovascular injury. Although the first 30 days is an important early postoperative time period, studies that track longer-term outcomes would yield a more comprehensive analysis. Complications that can occur after the 30 days include loosening of implants, malunion/nonunion, late infections, hardware failure, and fracture.^17,21^ Additionally, as the NSQIP database does not record thromboembolic risk factors such as prior contraceptive use or inherited thrombophilias, it was not possible to account for those variables in a logistic regression model. Another limitation is the lack of detailed description for each surgical procedure since all included patients were identified via CPT codes. As a result, different surgical techniques, such as opening-wedge and closing-wedge HTO, and detailed information on implants and imaging guidance−assisted procedures could not be collected. Further, since the data in the current study were from multiple institutions, there is heterogeneity in practice environment, sterilization technique, and surgical indications that may influence the results.

## Conclusion

There were no differences in 30-day VTE, UTI, transfusion, deep SSI, and reoperation rates for HTO versus UKA in the treatment of knee OA. HTO was associated with a higher rate of superficial SSI compared with UKA. These findings serve to guide clinicians in counseling patients regarding the early risks after HTO and UKA.
